# Innovative Design Methodology for Patient-Specific Short Femoral Stems

**DOI:** 10.3390/ma15020442

**Published:** 2022-01-07

**Authors:** William Solórzano-Requejo, Carlos Ojeda, Andrés Díaz Lantada

**Affiliations:** 1 Product Development Laboratory, Department of Mechanical Engineering, Universidad Politécnica de Madrid, C/José Gutiérrez Abascal 2, 28006 Madrid, Spain; 2 Mechanical Technology Laboratory, Department of Mechanical and Electrical Engineering, Universidad de Piura, Piura 20009, Peru; carlos.ojeda@udep.edu.pe or

**Keywords:** biomechanics, hip replacement, short stems, custom-made medical devices, strain shielding, finite element analysis

## Abstract

The biomechanical performance of hip prostheses is often suboptimal, which leads to problems such as strain shielding, bone resorption and implant loosening, affecting the long-term viability of these implants for articular repair. Different studies have highlighted the interest of short stems for preserving bone stock and minimizing shielding, hence providing an alternative to conventional hip prostheses with long stems. Such short stems are especially valuable for younger patients, as they may require additional surgical interventions and replacements in the future, for which the preservation of bone stock is fundamental. Arguably, enhanced results may be achieved by combining the benefits of short stems with the possibilities of personalization, which are now empowered by a wise combination of medical images, computer-aided design and engineering resources and automated manufacturing tools. In this study, an innovative design methodology for custom-made short femoral stems is presented. The design process is enhanced through a novel app employing elliptical adjustment for the quasi-automated CAD modeling of personalized short femoral stems. The proposed methodology is validated by completely developing two personalized short femoral stems, which are evaluated by combining in silico studies (finite element method (FEM) simulations), for quantifying their biomechanical performance, and rapid prototyping, for evaluating implantability.

## 1. Introduction

Hip arthroplasty is a surgical procedure in which the total or partial replacement of the hip joint is performed, using an artificial device, the hip/femoral prosthesis, which is a mechanical implant that replaces the joint primarily for two typical situations: to reduce pain and improve joint mobility due to progressive wear caused by osteoarthritis, or fracture of the femoral neck due to trauma or osteoporosis [1]. 

Koch’s femoral model, which defines two different sets of stress lines with compressive loads along the medial side and tensile loads on the lateral one, has been used to design stems for total hip replacement (THR). Consequently, conventional stems use the medial side as the support, also called calcar, because bone tissue is more resistant to compression than to tension and its use reduces the likelihood of fracture. However, Koch’s model does not accurately describe the biomechanics of the femur because it ignores muscle action; the forces generated by the iliotibial band and the vastus lateralis–gluteus medius complex create a tension band effect that converts the tensile stresses of the lateral femoral column into compressive ones [2,3]. 

Thus, it is proven that the cortical bone of the femur is subjected to compressive stresses in normal function, in accordance with its histological characteristics. This rethinking of the mode of load transfer on the entire proximal femur revolutionizes the design requirements of an anatomic cementless femoral implant [2].

The fixation of the cementless stem depends on the natural adherence between bone and stem; when properly adhered, the implant is stable. However, there is short- (primary) and long-term (secondary) stability. Primary stability depends on the tight insertion of the stem into the femoral canal; mechanically, it is quantified through the relative displacements that occur at the bone–stem interface [4]. The secondary stability is achieved through the bone ingrowth on its surface. This process is known as osseointegration; for this reason, the stem has a porous and textured coating. In addition, the material from which they are made should be biocompatible and not reactive to bone formation. 

The ideal stem should restore the physiological load transfer of the femur; unfortunately, after its insertion, the load pattern is modified. Consequently, the natural response of the bone to the conventional (stiffer) stem is proximal bone resorption and distal bone formation, due to which the phenomenon of stress/strain shielding (SS) arises, which occurs when part of the loads is taken up by the stem and prevented from reaching the femur, resulting in decreased bone, reducing the implant support and increasing the risk of loosening and fracture. The effects of aseptic loosening and micro-displacement can cause difficulties for patients when performing daily activities. If this situation is prolonged, it can cause a lot of pain and revision surgery is likely to be performed; however, the bone surrounding the removed femoral component has less bone stock; therefore, the new implant must be longer and thicker to be stabilized. However, strain shielding may occur again; therefore, this phenomenon should be eliminated [5]. 

Short stems were designed as an alternative to conventional implants to preserve the proximal bone stock. Calcar loading with lateral flare stems [6,7,8], a type of short implant, is attributed to Santori et al. [2,9], whose idea was to eliminate the diaphyseal part of the conventional stem because it causes shielding, and Jasty et al. [10] reported that it became unusable once the implant was stabilized and bone ingrowth occurred. 

Therefore, they deduced that, if this was true for a conventional prosthesis, it had to be true also for a stem that relies on a wide lateral flare for initial stability. It is recommended that the prosthesis be implanted initially in patients with good bone quality and normal anatomy. Contraindications for use are hip dysplasia, severe osteoporosis and previous hip osteotomies.

The objective of this stem is a physiological distribution with a proximal load transfer from the implant to the femur, restoring its biomechanics. In addition, by reducing the invasion of the femur, it may preserve good irrigation and nutrition, which would benefit the cellular action, and therefore the bone remodeling, and would consequently decrease the risk of avascular necrosis. Since the results of the implant are satisfactory both for stability and fixation, they would be even more so if it were personalized, eliminating the risks that are a consequence of errors in surgery due to poor selection and/or adaptation of the implant to the femoral cavity. 

This article seeks to rethink the design methodology of customized hip prostheses, optimizing the stems for calcar loading by employing lateral flare stems, hereinafter referred to as short stem. The article describes how to obtain the virtual model of the proximal femur, and then explains the development of a novel elliptical fitting app that allows the morphological evaluation of the femur (geometric parameters and femoral cavity). Consequently, based on the morphology and the surgical procedure, the short stem is designed. Finally, the finite element method (FEM) is employed to verify the biomechanical advantages described and to validate the designed short stems. 

## 2. Materials and Methods

### 2.1. Virtual Model

**Figure 1:**
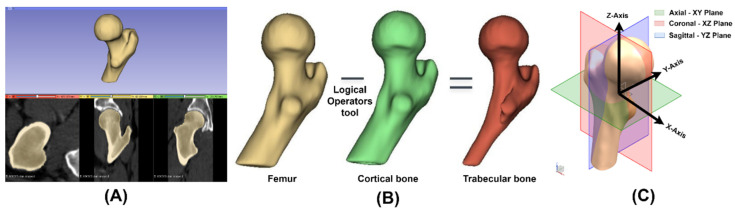
(**A**) Segmentation of the right femur using 3D Slicer^®^. (**B**) Process to obtain the trabecular bone. (**C**) Femoral coordinate system.

Virtual models of the proximal femur were used, obtained by downloading the CT scans of two male patients from the open-source virtual library *“The Cancer Imaging Archive*” with references TCGA-VP-A878 [11] and Pelvic-Ref-009 [12] corresponding to the geometric case 1 (GC1) and 2 (GC2). The medical images had a slice thickness of 2 and 3 mm in the axial plane, and 0.909 and 1 mm in the coronal and sagittal planes for GC1 and GC2, respectively, each image being 512 × 512 pixels. Both CT scans were imported into 3D Slicer^®^ 4.10.2 (https://www.slicer.org, accessed on 3 January 2022) to segment the right femur and its cortical part (Figure 1A) using the threshold, level tracing, paint, erase and smooth tools. Then, the trabecular part was obtained through the logical operation of subtraction between the femur and its cortical bone (Figure 1B). The 3D Slicer^®^ allowed us to export the segmentation of the femur, cortical and trabecular bone as meshes in STL format, and these files were imported into Meshmixer^®^ 3.5 (Autodesk Inc., Mill Valley, CA, USA) for inspection, repair and smoothing. Finally, the virtual models were processed as solids in NX^®^ 10 (Siemens PLM Software Solutions, Plano, TX, USA), matching their coordinate system with the femur’s coordinate system so the axial, coronal and sagittal planes were the XY, XZ and YZ planes, respectively (Figure 1C). 

### 2.2. Elliptical Adjustment App

**Figure 2:**
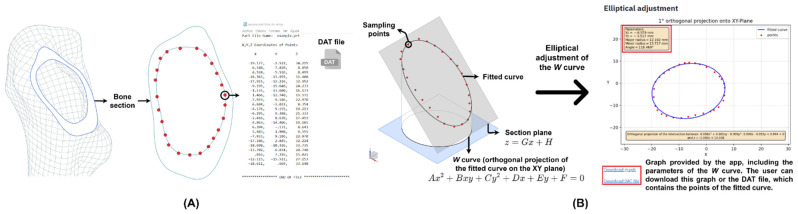
(**A**) Bone section sampling and DAT file. (**B**) Representation of the fitted curve and the elliptical adjustment of its orthogonal projection.

Trapezoidal, oval, elliptical and circular cross-sections have been used for the design of femoral stems. Previous studies [13,14] determined that the elliptical section produces a good stress distribution along the stem, allows its primary stability and improves its adaptability to different bone sections with changes in shape and size. Therefore, using Streamlit^®^, an open-source Python^®^ library, an elliptical adjustment app was created to obtain the ellipse that best fits the bone section (https://github.com/solor5/elliptical_adjustment_app/blob/main/app.py, accessed on 3 January 2022). To utilize the application, the user must sample the bone section to be adjusted by employing the NX^®^ point tool, and the coordinates of each point are exported in a DAT file (Figure 2), which is inserted into the app file uploader. The mathematical fundamentals that enable the elliptical fitting are explained below.

As a consequence of the position of the femur in NX^®^ (Figure 1C), the bone section is located in an oblique plane perpendicular to the XZ; therefore, the X and Y coordinates of the points in the DAT file (p) allow the elliptical adjustment of the orthogonal projection of this section (Figure 2A). This conic (W) is represented by an implicit second-order polynomial (Q), defined by a vector of coefficients ($v={\left[A\;B\;C\;D\;E\;F\right]}^{T}$):

(1) $$W\left(v\right)=\{p\in \;{\mathbb{R}}^{2}\;|\;Q\left(p,v\right)=0\}$$

(2) $$Q\left(p,v\right)=\left[x^{2}\;\;\;\;xy\;\;\;\;y^{2}\;\;\;\;x\;\;\;\;y\;\;\;\;1\right]\;\;\cdot \;\;\left[\begin{array}{c} A\\ B\\ \;\;C\;\;\\ D\\ E\\ F \end{array}\right]=Ax^{2}+Bxy+Cy^{2}+Dx+Ey+F=0$$

If $P=\left\{p_{1},\;p_{2},\;\ldots ,\;p_{n}\right\}$ is the set of points obtained from sampling the orthogonal projection of the bone section, it only includes the X and Y coordinates of the DAT file loaded in the app. The vector of coefficients must be adjusted to P; for this purpose, the algebraic distance ($D_{A}$) was used. This distance is widely employed because it simplifies the calculations and needs less computational resources [15]. Mathematically, it is obtained by replacing the coordinates of a point $p_{i}=\left(x_{i},y_{i}\right)$ in the Q polynomial; hence, if $p_{i}$ belongs to the ellipse, its distance will be 0.

(3) $$D_{A}\left(p_{i},\;W\left(v\right)\right)=Q\left(p_{i},v\right)=Ax_{i}^{2}+Bx_{i}y_{i}+Cy_{i}^{2}+Dx_{i}+Ey_{i}+F$$

The least squares technique optimizes the fit by minimizing the square of the algebraic distance between the P points and the W curve, and it can be expressed as the squared norm of the product between the design matrix $D_{P}$, which contains information of P, and the v vector.

(4) $$D_{p}=\left[\begin{array}{c} x_{1}^{2}\;\;\;x_{1}\;\;\;y_{1\;\;\;}y_{1}^{2}\;\;\;x_{1}\;\;\;y_{1}\;\;\;1\\ x_{2}^{2}\;\;\;x_{2}\;\;\;y_{2\;\;\;}y_{2}^{2}\;\;\;x_{2}\;\;\;y_{2}\;\;\;1\\ \;\vdots \;\;\;\;\;\vdots \;\;\;\;\;\;\vdots \;\;\;\;\;\;\vdots \;\;\;\;\;\;\vdots \;\;\;\;\;\;\vdots \;\;\;\;\;\vdots \\ x_{n}^{2}\;\;\;x_{n}\;\;\;y_{n}\;\;\;y_{n}^{2}\;\;\;x_{n\;\;\;}y_{n}\;\;\;1 \end{array}\right]$$

(5) $$min\overset{n}{\underset{i=1}{\sum }}D_{A}{\left(P,\;W\left(v\right)\right)}^{2}=min\overset{n}{\underset{i=1}{\sum }}Q{\left(P,v\right)}^{2}=min\;\|D_{P}v{\|}^{2}$$

To avoid the trivial solution of $v={\bar{0}}_{6}$, the vector of coefficients is bounded [15]. Paton [16] analyzed the chromosome shape using a conic fit with a constraint of $\|v{\|}^{2}=1$, avoiding all coefficients being zero. Therefore, using this constraint, the solution can be an ellipse, hyperbola or parabola; however, because the bone sections, especially in the diaphyseal part, have an elliptical shape, the conic provided by the app will be an ellipse. The Lagrange multipliers allowed us to minimize the distance considering the constraint. Consequently, L is the Lagrange function to be optimized.

(6) $$L=\|D_{P}v{\|}^{2}-\lambda \left({\left\|v\right\|}^{2}-1\right)=v^{T}D_{P}^{T}D_{P}v-\lambda \left(v^{T}v-1\right)$$

Equating the gradient of L with respect to v to 0 for minimizing the function gives:

(7) $$\nabla_{v}L=0\;\Leftrightarrow \;2D_{P}^{T}D_{P}v-2\lambda v=0$$

(8) $$D_{P}^{T}D_{P}v=\lambda v$$

The optimization leads to the eigenvector problem; then, λ and v must be an eigenvalue and an eigenvector of $D_{P}^{T}D_{P}$. If $D_{P}^{T}D_{P}v=\lambda v$, Equation (5) will be:

(9) $$min\|D_{P}v{\|}^{2}=min\;v^{T}D_{P}^{T}D_{P}v=min\;\lambda \|v{\|}^{2}=min\;\lambda$$

As a result, the coefficient vector (v) that minimizes the algebraic distance will be the eigenvector of $D_{P}^{T}D_{P}$ corresponding to the smallest eigenvalue (λ). Once v is found, Q is defined; however, although CAD programs allow users to enter functions to draw a curve, this operation is often tedious. Therefore, the W ellipse can be defined with five parameters: the coordinates of its center ($x_{c},y_{c}$), the largest (R) and smallest (r) radius and the angle (α) that rotates the curve counterclockwise. The coefficients of v are renamed:

(10) $$v={\left[ABCDEF\right]}^{T}={\left[a^{\prime }\;2b^{\prime }\;c^{\prime }\;2d^{\prime }\;2e^{\prime }\;f^{\prime }\right]}^{T}$$

The five parameters can be calculated using the following equations [17]:

(11) $$x_{c}=\frac{c^{\prime }d^{\prime }-b^{\prime }e^{\prime }}{{b^{\prime }}^{2}-a^{\prime }c^{\prime }}$$

(12) $$y_{c}=\frac{a{}^{\prime }e{}^{\prime }-b{}^{\prime }d{}^{\prime }}{b{{}^{\prime }}^{2}-a{}^{\prime }c{}^{\prime }}$$

(13) $$R=\sqrt{\frac{2\left(a{}^{\prime }e{{}^{\prime }}^{2}+c{}^{\prime }d{{}^{\prime }}^{2}+f{}^{\prime }b{{}^{\prime }}^{2}-2b{}^{\prime }d{}^{\prime }e{}^{\prime }-a{}^{\prime }c{}^{\prime }f{}^{\prime }\right)}{\left(b{{}^{\prime }}^{2}-a{}^{\prime }c{}^{\prime }\right)\left[\sqrt{{\left(a{}^{\prime }-c{}^{\prime }\right)}^{2}+4b{{}^{\prime }}^{2}}-\left(a{}^{\prime }+c{}^{\prime }\right)\right]}}$$

(14) $$r=\sqrt{\frac{2\left(a{}^{\prime }e{{}^{\prime }}^{2}+c{}^{\prime }d{{}^{\prime }}^{2}+f{}^{\prime }b{{}^{\prime }}^{2}-2b{}^{\prime }d{}^{\prime }e{}^{\prime }-a{}^{\prime }c{}^{\prime }f{}^{\prime }\right)}{\left(b{{}^{\prime }}^{2}-a{}^{\prime }c{}^{\prime }\right)\left[-\sqrt{{\left(a{}^{\prime }-c{}^{\prime }\right)}^{2}+4b{{}^{\prime }}^{2}}-\left(a{}^{\prime }+c{}^{\prime }\right)\right]}}$$

(15) $$\alpha =\{\;\begin{array}{c} 0;if\;b{}^{\prime }=0\;and\;a{}^{\prime }<c{}^{\prime }\\ \frac{\pi }{2};if\;b{}^{\prime }=0\;and\;a{}^{\prime }>c{}^{\prime }\\ \frac{arctan\left(\frac{2b{}^{\prime }}{a{}^{\prime }-c{}^{\prime }}\right)}{2};if\;b{}^{\prime }\neq 0\;and\;a{}^{\prime }<c{}^{\prime }\\ \frac{\pi }{2}+\frac{arctan\left(\frac{2b{}^{\prime }}{a{}^{\prime }-c{}^{\prime }}\right)}{2};if\;b{}^{\prime }\neq 0\;and\;a{}^{\prime }>c{}^{\prime } \end{array}$$

The fitted curve of the bone section is the intersection between an elliptical cylinder, with the W curve as its directrix and the Z-axis as its generatrix, and the section plane z=Gx+H (Figure 2B), where the constants (G, H) are fitted using linear regression from the X and Z coordinates of each point of the DAT file. There are two ways to export the fitted curve from the app to NX^®^. The first one allows the user to obtain the points of the curve in DAT format by clicking on *Download DAT file* (Figure 2B), and then import these points to NX^®^ and, with the spline tool, obtain the fitted curve. Likewise, the application provides a graph of the W curve containing the parameters that define it ($x_{c},y_{c},\;R,\;r$,α); these are introduced in the NX^®^ ellipse tool and W is projected to the section plane to obtain the fitted curve (Figure 2B). The elliptical adjustment app allows the user to download the graph of the W curve (Figure 2B) and provides a 3D view of the fitted curves because the app can make several adjustments at the same time.

### 2.3. Morphological Study

Morphological study of the proximal femur is essential because it is the region that undergoes long-term bone resorption in most state-of-the-art implants [18]. During preoperative planning of THR, the surgeon chooses a suitable stem from among the prostheses manufactured in advance. For this purpose, he/she evaluates the patient’s morphology using radiographs; however, the femur has specific and individual characteristics, and this technique does not provide detailed information about the femoral cavity, so the chosen stem may fill it poorly or exceed its dimensions, causing periprosthetic fractures. 

In addition, the geometric parameters neck–shaft angle, anteversion and offset would be inadequate, which could result in a dislocated stem [19,20,21,22,23,24]. The three-dimensional femoral model obtained from the CT scan (Figure 1A) provides more accurate information that allows the morphological study of each patient because it is essential for the customized design of cementless stems, since precise dimensions of the femoral canal guarantee mechanical stability and avoid SS [25]. 

#### 2.3.1. Neck–Shaft and Mechanical Angle

**Figure 3:**
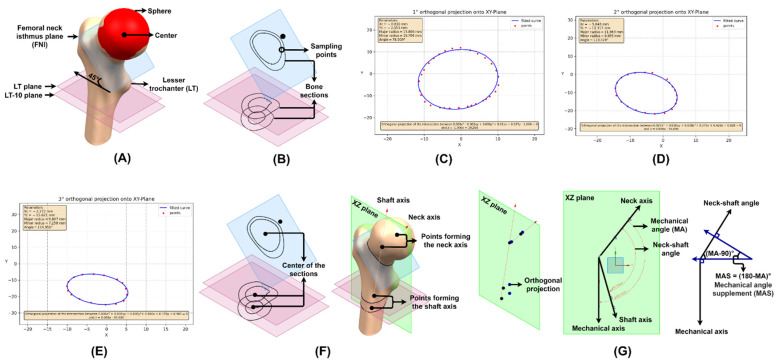
Steps to measure the neck–shaft and mechanical angle. (**A**) Estimation of the femoral head with a sphere and location of the FNI, LT and LT-10 planes. (**B**) Sampling of bone sections. The adjustment made by the app for sections (**C**) FNI, (**D**) LT and (**E**) LT-10. (**F**) Neck and shaft axis. (**G**) Neck–shaft and mechanical angle.

To measure the neck–shaft angle, modifications of the techniques described by Wang et al. [26] and Zhang et al. [27] were used. Previously, the femoral head was simulated as a sphere; thus, its centers are coincident. If this estimation is not possible due to the fracture of the femoral neck, the acetabulum can be used to define the sphere. Three reference planes are located: the first at the femoral neck isthmus (FNI), a plane parallel to the XY plane and rotated 45° clockwise with respect to the X-axis—45° is the supplement of the average neck–shaft angle according to the study by Gilligan et al. [28]; the second and third planes are located at the end of the lesser trochanter (LT) and 10 mm (LT-10) below and both are parallel to the XY plane (Figure 3A).

The proximal femur is cut through all three planes, generating bone sections. As described in the section *Elliptical adjustment app* (Section 2.2), the center of the bone section can be found by sampling it with NX^®^ (Figure 3B). The fit performed by the app for the FNI, LT and LT-10 sections is shown in Figure 3C–E, respectively. The femoral neck axis passes through the centers of the sphere and the FNI section, and the shaft axis passes through the centers of the LT and LT-10 sections (Figure 3F). Both axes are orthogonally projected on the XZ plane and the angle between them is the neck–shaft angle. The mechanical axis is parallel to the Z-axis, and the angle between the neck and the mechanical axes is the mechanical angle (Figure 3G). Therefore, the neck–shaft and mechanical angle (MA) for GC1 are 126.4° and 141.9°, and for GC2 are 133.1° and 143°.

The neck–shaft angles of GC1 and GC2 are within the normal range of 90° to 135°; if the inclination is greater than 125°, it is called *coxa valga*, and if it is less than 120°, *coxa vara* [29]. If the stem selected by the orthopedist or designed by the engineer alters the patient’s neck–shaft angle, valgus or varus position, a muscular imbalance is generated and, as a consequence, affects the load to which the joint is subjected after THR, favoring the loosening of the implant [30,31,32].

#### 2.3.2. Anteversion

**Figure 4:**
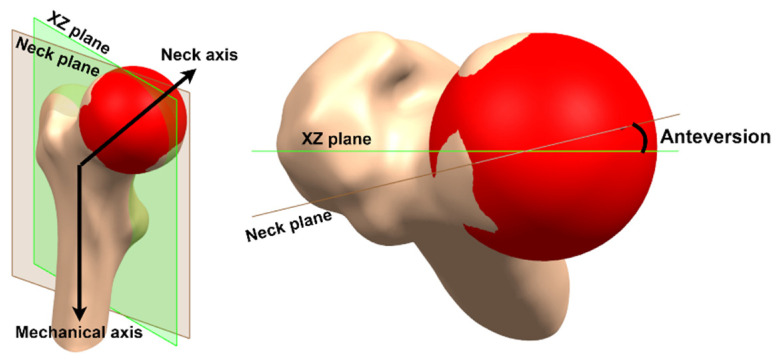
Anteversion.

Yadav et al. [33] measure femoral anteversion three-dimensionally as the angle between the condylar plane, formed by the condylar and neck axes, and the femoral neck plane, composed of the neck and shaft axes. However, the virtual model of the proximal femur (Figure 1) does not include the condyles, and a new strategy to quantify anteversion is proposed, which consists of taking the XZ plane as a reference and redefining the femoral neck plane as the one formed by the neck and mechanical axes, since both planes are formed by an axis parallel to Z, favoring the measurement of anteversion: the angle between the new femoral neck plane and the XZ plane (Figure 4). The approximate anteversion for GC1 and GC2 is 13.5° and 3.6°, respectively.

Anteversion aims to restore the femoral center of rotation [34]. Its reduction leads to increased external rotation of the leg, increases torsional moments on the prosthesis [35,36,37] and may be associated with an increased risk of loosening [38]. Moreover, it has a strong influence on hip contact forces [39]; therefore, the correct anteversion angle allows an optimal range of motion with minimal risk of instability [40,41].

#### 2.3.3. Offset

The offset is the perpendicular distance between the shaft axis and the center of the femoral head. Because the femoral head was simulated as a sphere to measure the neck–shaft angle (Figure 3A), it has implicit offset information, so it is not necessary to quantify the offset on the condition that the sphere is used in the custom design. This parameter improves physical function, increases hip stability, maintains postoperative pelvic balance and minimizes the risk of dislocations [31,42,43]. Several studies have shown that an increase in offset correlates with a reduced neck–shaft angle, increased range of motion, increased lever arm and abductor strength. If not restored, it increases the reactive force of the joint, consequently causing wear and leading to implant failure [42,43,44,45].

#### 2.3.4. Femoral Cavity

The customized stem design determines the areas of contact with the cortical bone, which results in differences in biomechanics and fixation between implants. The goal is to achieve initial stability through fixation with adequate bone contact [46], hence indicating the importance of studying the femoral cavity, as it geometrically delimits the dimensions of the stem and prevents early loosening and periprosthetic fractures. In addition, unlike the geometric parameters of the femur, which correlate with each other, the femoral cavity has highly variable characteristics specific to each person, so it is not proportional to the external femoral geometry. The study of the femoral cavity for the design of conventional stems consists of orthogonal cuts that section the bone [1]. However, the cutting planes will host the sketches that will compose the stem, which is obtained from the interpolation of them. 

**Figure 5:**
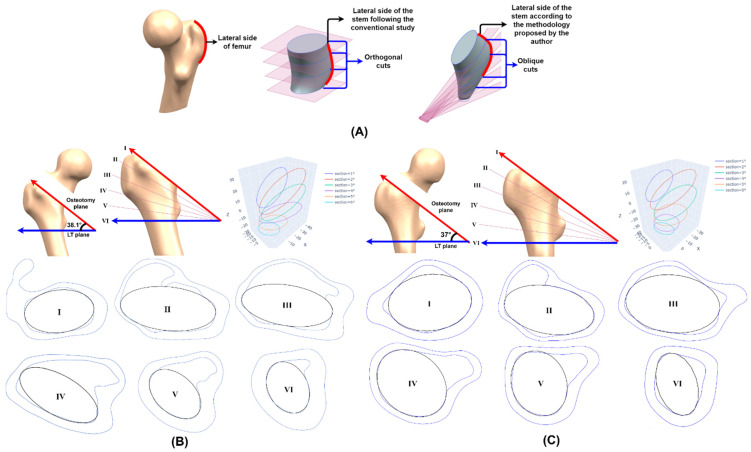
(**A**) Methodologies to study the femoral cavity. Femoral cavity analysis of (**B**) GC1 and (**C**) GC2.

As shown in Figure 5A, if the conventional analysis is performed, the result does not mimic the lateral side of the proximal femur, increasing the SS because the biomechanics are not restored, since this methodology is optimized to adapt the contact between implant and bone in the calcar and the femoral diaphysis. For this reason, another technique is needed to study the cavity and design the personalized short stem.

To replicate the curvature of the lateral side, it was necessary to create an arch whose origin generates oblique planes that allow the study of the canal and the design of the stem that adapts to it. To generate the arch, the LT plane was used (Section VI, Figure 5), and then an oblique plane (Section I, Figure 5) was placed below the FNI plane because, according to the study by Solórzano et al. [18], this is the area with the highest risk of fracture of the proximal femur. Finally, the arc created from both planes, whose interior angle is the mechanical angle supplement (MAS) (Figure 3G and Figure 5), was divided into five equal parts, producing the planes II, III, IV and V (Figure 5). 

It is possible to obtain more study planes by dividing the arch into more parts; however, the design becomes more complex and the stem less organic.

From the oblique planes, the bone sections used to study the cavity were obtained, and each one was sampled following the procedure described in the *Elliptical adjustment app*; see Section 2.2. The app provided the three-dimensional scheme of the fitted curves and the individual fitting graph of each section, which contained the ellipse parameters and allowed the import of the fitted curve to NX^®^ to check that it is properly adapted to the original bone section (Figure 5). These fitting curves constrained the stem geometry and allowed the study of its implantability.

### 2.4. Custom Design

Gómez-García et al. [47] mentioned that, in general, the short stem design has five basic defined characteristics: the anatomical region they occupy, geometric characteristics of the design, areas where stress transmission occurs, osteotomy and insertion. The short stem occupies and transmits stresses toward the metaphysis, due to which it is known as a metaphyseal stem. Therefore, throughout this section, geometry, osteotomy and insertion are integrated into the custom design of the short stem. As mentioned in the *Morphological study* (Section 2.3), the three femoral parameters play an important role in muscle action and range of motion; therefore, their preservation is crucial in order not to alter the femoral biomechanics.

#### 2.4.1. Osteotomy

This is the procedure that repairs damaged joints by cutting and remodeling the bones. In THR, its role is to remove the femoral neck to place a stem inside its cavity and remodel the acetabulum to align with the implant and create an artificial joint that restores the patient’s mobility. Hereafter, the term osteotomy is used to refer to the femoral neck removal. Dimitriou et al. [48] determined that the cutting plane, called the osteotomy plane, affects the implantation section, the bone section resulting from the removal of the neck through which the prosthesis enters in the cavity (I Section, Figure 5), and the postoperative position of the non-customized femoral stem altering the neck–shaft angle and anteversion due to the complex morphology of its proximal canal. Therefore, they suggest that the osteotomy be optimized considering the alignment of the stem that restores the femoral mechanical response, to avoid generating a muscular imbalance that accelerates loosening. However, in customized implants, this is achieved through individual analysis and design. Consequently, its role is the evaluation of the implantability, since the design of the personalized stem must guarantee the correct interaction between bone and implant (fit) and be able to enter through the implantation section (filling), to prevent fractures during surgery. Recalling the subsection *Femoral cavity* (Section 2.3.4), the I plane was located below the FNI and the angle that it formed with the LT plane or VI section was the MAS; this occurs because the I plane is the osteotomy plane and must consider a cutting zone below the fracture, which would occur in the FNI, and restore femoral parameters such as the neck–shaft angle through the mechanical one (Figure 5).

#### 2.4.2. Insertion

**Figure 6:**
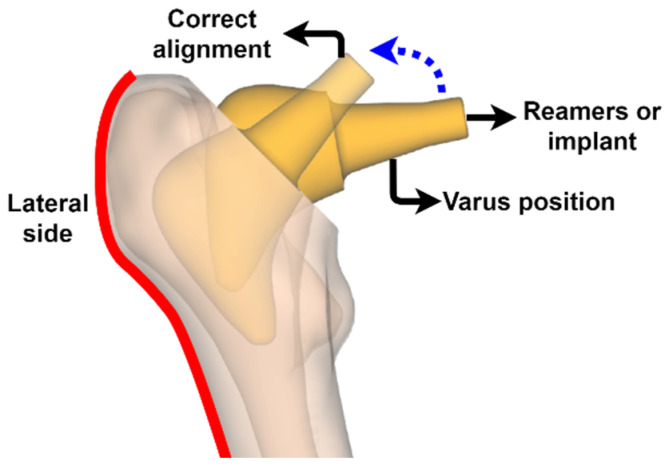
“Round the corner” technique.

The custom short stem design is characterized by mimicking the curvature of the lateral side of the proximal femur (Figure 5A). This lateral widening requires a new implantation method to achieve femoral reaming, which consists of gradually opening the cavity using calibrated elements similar to the stem until the appropriate size is achieved for insertion, while respecting the greater trochanter and the gluteal muscles. This technique has been called “round the corner” and is possible due to the absence of the distal part of the stem [2,9]. “Round the corner” requires that the reamers and final implant are first inserted in the varus position and then progressively tilted into the correct alignment while descending the femoral metaphysis (Figure 6). 

This technique facilitates the use of minimally invasive approaches such as MicroHip [49], but precludes the use of intramedullary guides and may also result in a varus position when the tip of the stem touches the lateral side of the femur, contributing to a possible fracture, so the use of fluoroscopy during insertion is advisable [50].

#### 2.4.3. Implantability

**Figure 7:**
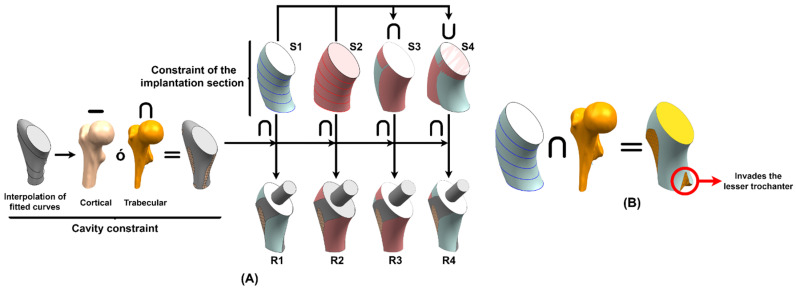
Assessment of implantability (**A**) without and (**B**) with the trabecular bone.

The designers, despite having the virtual model, often do not consider in the design process the osteotomy and the insertion method, key aspects that determine whether the customized prosthesis is implantable or not. Consequently, a methodology is proposed to study implantability by ensuring that the prosthesis adapts to the canal and its insertion is possible. From a geometric point of view, to use the “round the corner” technique, the limits of the implant sections must be projections of the implantation curve on the planes used in the cavity analysis (Figure 5), due to the rotation performed to place the stem in the correct alignment (Figure 6). The orthogonal projection of the implantation or osteotomy curve can be of two types: the first consists of projecting the I section on the oblique planes (S1); in the second, the I curve is projected on the II plane, and the result is projected on the III plane and so on until reaching the IV plane (S2). Interpolating the generated curves, two solids are formed; however, not only these bodies must be evaluated, but the intersection (S3) and union (S4) of both must also be included in the implantability analysis. These four solids represent the **constraint of the implantation section** (Figure 7A). 

However, the **cavity constraint** should be included in the analysis. As in the previous case, the adjusted curves obtained from the study of the femoral canal are interpolated, forming a solid that approximates the patient’s cavity; therefore, it is necessary to rectify areas of overestimation, which invade the cortical part of the femur, intercepting it with the trabecular bone or subtracting the cortical one, achieving, as a result, the maximum volume of the customized stem (Figure 7A). Both constraints must be considered to ensure the implantability of the prosthesis; therefore, the solids, which are a physical representation of the constraints generated by the patient’s cavity and the implantation section, have to be intercepted. As a result, four regions are produced, R1, R2, R3 and R4, which are a consequence of the intersection of S1, S2, S3 and S4 with the cavity constraint (Figure 7A).

Regarding the cavity constraint, the question may arise as to why the trabecular bone, which contains exact information about the patient’s cavity, is not used directly; this is because, when intercepting the trabecular bone with the constraint of the implantation section, the result invades the lesser trochanter, which, according to the study of Solórzano et al. [18], is a moderately critical area of the proximal femur (Figure 7B). Therefore, fitted curves that adapt to the femoral canal, and do not invade the lesser trochanter, allow proper implant design, avoiding periprosthetic fractures.

**Figure 8:**
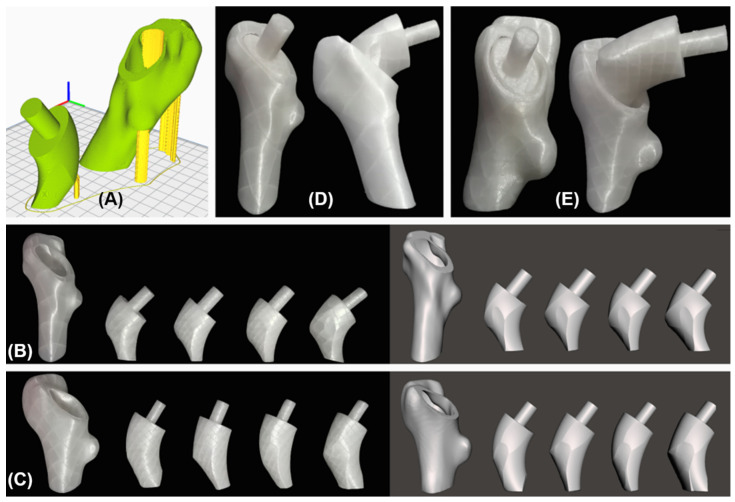
Prototype fabrication. (**A**) Planning using Ultimaker Cura^®^. PLA prototypes with their respective STLs for (**B**) GC1 and (**C**) GC2. Evidence of experiments performed for (**D**) GC1 and (**E**) GC2 demonstrating the insertion of regions 1, 2 and 3 (left), but not 4 (right).

To test the implantability, the four regions and the cortical part already included in the osteotomy for GC1 (Figure 8B) and GC2 (Figure 8C) were fabricated to imitate the “round the corner” technique, certifying that the regions enter through the I section and fit the cavity properly. 

Therefore, using fused material deposition printing, PLA prototypes were produced from the STL files of the solids, which were laminated in Ultimaker Cura 4.8.0^®^ (Ultimaker, Geldermalsen, Netherlands; Figure 8A) and manufactured using the Ender 3 Pro^®^ (Creality, Shenzhen, China) printer.

The results showed, for both geometric cases, that regions 1, 2 and 3 are implantable solids; therefore, the customized stem was designed from them; however, region 4 is not implantable because it did not enter through the cavity (Figure 8D,E). Now, the choice of two geometric cases with different femoral morphology makes sense since it gives reliability to the conclusions obtained from experimentation; however, to generalize this behavior, further testing with other patients is needed. Moreover, to emphasize that this study is possible thanks to the new methodology proposed to study the femoral cavity since, had the conventional technique been used, the restriction of the implantation section would be an elliptical cylinder. As a result, the implantable solid would not adapt to the lateral side of the femur, which would impair its biomechanics after surgery.

#### 2.4.4. Stem

The implantable regions, because they adequately fit and fill the patient’s cavity and enter through the implantation section, were the stem of the customized short implant. However, because of the Boolean operations performed, they were not uniform (Figure 8), hindering bone ingrowth; therefore, they were smoothed, preserving their shape using the Meshmixer^®^ smooth tool with a smoothing scale of 50, and, to facilitate its insertion through the femoral canal, the edge of the VI section was rounded by 5 mm.

#### 2.4.5. Neck and Receiving Taper

Mimicking European standards, the custom stem taper was 12/14 since, according to the study by Morlock et al. [51], this receiving taper is the most commonly used in that continent. The 12/14 model is defined by a proximal diameter of 12 mm, distal of 14 mm and a height of 20 mm, resulting in a taper angle of 5°43’30’’. 

**Figure 9:**
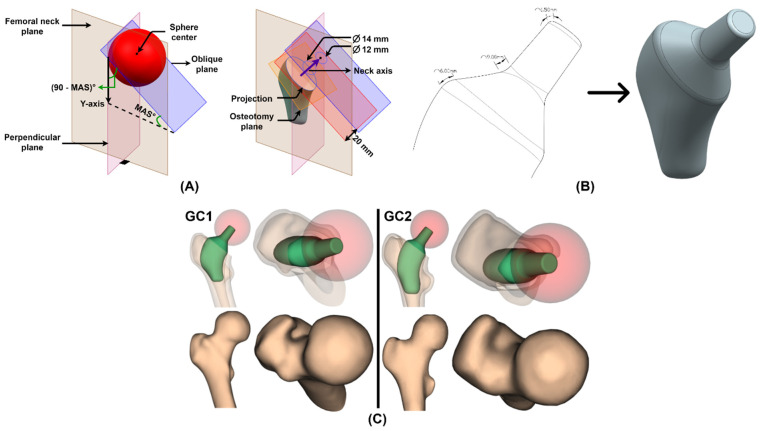
Neck and receiving taper (**A**) sketches and (**B**) solid. (**C**) Comparison between implanted and intact femur for GC1 and GC2.

To model the taper, a plane perpendicular to the femoral neck plane was defined and, to preserve the neck–shaft angle in the design, it was rotated (90-MAS)° counterclockwise with respect to the Y-axis. In addition, following the recommendation of Wen-Ming et al. [24], it was placed at the middle of the sphere, which approximates the femoral head, obtaining an oblique plane where the sketch of the 12 mm circumference was drawn. To maintain the height of the cone, a plane 20 mm below the oblique plane was positioned and the sketch of the 14 mm circumference was drawn following the direction of the femoral neck axis. For the neck, the initial curve of the stem was needed, which was obtained by projecting the first section onto the osteotomy plane. This whole process is illustrated in Figure 9A. 

Based on the drawn curves, which form the neck and the receiving taper, a solid was obtained and integrated into the stem through the Boolean union operation; to avoid stress concentration, the edges of the curves were rounded as shown in Figure 9B. The described process was repeated for each region (R1, R2 and R3) to obtain the final custom stem designs (V1, V2 and V3). Once the design of the short stem has been completed with the described methodology that considers the patient’s features, such as his anatomy, and those that depend on the surgery—osteotomy and insertion—the adjustment, filling and implantability of the prosthesis are guaranteed. However, to carry out the design, elements of the femoral morphology study were used, such as the LT plane, the MAS, the femoral neck axis and plane and the sphere that fits the femoral head, thanks to which it was possible to restore the neck–shaft angle, anteversion and offset of each geometric case, as visualized in Figure 9C. Therefore, none of the proposed designs is expected to fail due to muscle imbalance, modification of the range of motion or impingement with the acetabulum. Another point in favor of this technique is its simplicity, since the designer does not need to manually define the implant–bone contact zones because the program created provides the curve that best fits the bone section guaranteeing primary stability, thus avoiding human error in the process. The next step was to perform the finite element analysis (FEA) to select which of the three options is the best stem for each geometric case.

### 2.5. Finite Element Model 

#### 2.5.1. Mesh

NX^®^ was used for performing FEA, employing its Nastran solver. There are two finite element models: intact and implanted femur. In the study of the intact femur, only the cortical and trabecular bone are involved; on the contrary, in the implanted femur, the two osteotomized bones and the customized stem interact. The cortical and trabecular bone for GC1 was meshed with an element size of 1.87 mm; for GC2, the size was 1.3 mm, both for the intact and implanted femur. The stems for each geometric case were meshed with an element size of 0.9 mm. All bodies used CTETRA 10 as the element. The selection of these element sizes and type is a consequence of the convergence analysis performed using the p-method and h-method with an admissible error of 2%, which considers the quality of the results and the speed of calculation. Because most of the remodeling processes occur in full osseointegration [52], the meshes are joined through the “surface-to-surface bonding” tool. 

#### 2.5.2. Bone Properties

The biomechanical behavior of bone is extremely complex due to its anisotropic and viscoelastic nature. However, it exhibits elastic behavior under usual mechanical conditions. The femur being a long bone, the analysis was performed considering the transversely isotropic properties of cortical bone and, according to the literature [53], it has been assumed that the trabecular bone presents a large-scale isotropy. Bone properties were estimated using the apparent density ($\rho_{app}$), which was obtained employing the “Segment Statistics” tool of 3D Slicer^®^ [18] and considering its relationship with the Hounsfield units (HU). Rho et al. [54] determined a linear relationship between HU and apparent density for the proximal femur:

(16) ρapp=1311000+1.067HU1000[gcm3]

The Young’s modulus of cortical bone in the longitudinal direction ($E_{z,cortical}$) and the stiffness of trabecular one ($E_{trabecular}$) were estimated using the equation described by Keyak et al. [55] and rectified by Schileo et al. [56]:

(17) $$E_{z,cortical}=E_{trabecular}=14,900{\left(0.6\rho_{app}\right)}^{1.86}\left[\mathrm{MPa}\right]$$

In addition, the Young’s modulus ($E_{x},\;E_{y}$) and shear modulus ($G_{yz},G_{zx}$) in the transverse direction, for cortical bone, were calculated using Pithioux’s laws [57]:

(18) $$E_{x}=E_{y}=0.6E_{z}\;$$

(19) $$G_{yz}=G_{zx}=0.25E_{z}$$

Poisson’s coefficients in the longitudinal ($\nu_{yz},\nu_{zx}$) and transverse ($\nu_{xy}$) directions of cortical bone were obtained from the literature, being 0.25 and 0.4, respectively [58]; the value of 0.3 for the Poisson’s coefficient (ν) of trabecular bone was taken from experimental data [59]. The shear modulus in the longitudinal direction ($G_{xy}$) of cortical bone [60,61] and the shear modulus (G) of trabecular bone were obtained from the following equations:

(20) $$G_{xy}=\frac{E_{x}}{2\left(1+\upsilon_{xy}\right)}$$

(21) $$G=\frac{E}{2\left(1+\nu \right)}$$

Table 1 summarizes the physical and mechanical properties of both bones for GC1 and GC2.

#### 2.5.3. Stem Properties

The material must be biocompatible to promote osseointegration, and the bone must grow close to the implant surface and fill the grooves or pores that have been deliberately introduced to firmly embed the stem and reduce the bone resorption, be immune and inert to corrosion by body fluids and tissues, be strong and ductile to withstand the mechanical demands of the patient’s daily activity, have low density, be light so as not to affect gait and not have magnetic properties, to perform a clinical evaluation after surgery using medical imaging such as MRI or CT [62,63,64,65].

Among the materials employed in the manufacture of femoral prostheses, the most used is Ti6Al4V because its Young’s modulus is close to that of bone and it has proven to be more biocompatible than stainless steel and cobalt–chromium–molybdenum [65]; it also meets the requirements mentioned above. Nevertheless, titanium implants are retained in bone by mechanical and chemical stabilization, as, through direct contact between calcium atoms and the titanium oxide surface, they create an inorganic interface, leading to osseointegration [46]; however, wear caused by friction between bone and implant liberates metal ions that react biologically with the body, including aluminum ions, which have been linked to the development of diseases such as Alzheimer’s and cytotoxicity caused by excessive concentrations of vanadium [66,67]. 

A substitute for Ti6Al4V may be the Ti alloy Ti-15Mo-2.7Nb-3Al-0.2Si, also known as Ti21S, because it reduces the aluminum content, eliminates vanadium, improving its cytotoxicity, and presents an extremely low Young’s modulus, good strength and ductility, excellent corrosion resistance and biocompatibility, which makes this material suitable for biomedical applications [68]. Additive manufacturing (AM) technologies allow the fabrication of specific and intricate patient geometries, reduce stiffness due to inherent porosity and roughness, have been shown to promote bone ingrowth and employ efficient material usage [69,70,71,72]. Therefore, Ti6Al4V ELI (extra low interstitials) and Ti21S were defined as the stem material for the FEA (Table 2) since both are used in the AM of femoral stems.

#### 2.5.4. Boundary Conditions

Solórzano et al. [18] studied the mechanical behavior of the proximal femur against the nine loads proposed by Bergmann et al. [74], including the ISO force [75], widely used to test femoral stems. They concluded that the representative loads that increase the risk of fracture are ISO and jogging; consequently, both were used to evaluate the differences between the biomechanics of the intact and implanted femur. The jogging load for the intact femur is only composed by the contact forces ($F_{X},\;F_{Y}$ and $F_{Z}$); however, when the stem is implanted, the moments that stress the fixation in the acetabulum appear; hence, the implanted femur is subjected to contact forces and frictional moments ($M_{X},\;M_{Y}$ and $M_{Z}$). This load depends on the body weight of each patient; nevertheless, in this study, the same load state (shown in Table 3) was used for GC1 and GC2 of our recently explained procedure [18], since, being equal, the boundary conditions allowed us to evaluate and compare the influence of the femoral morphology in the stem design.

**Figure 10:**
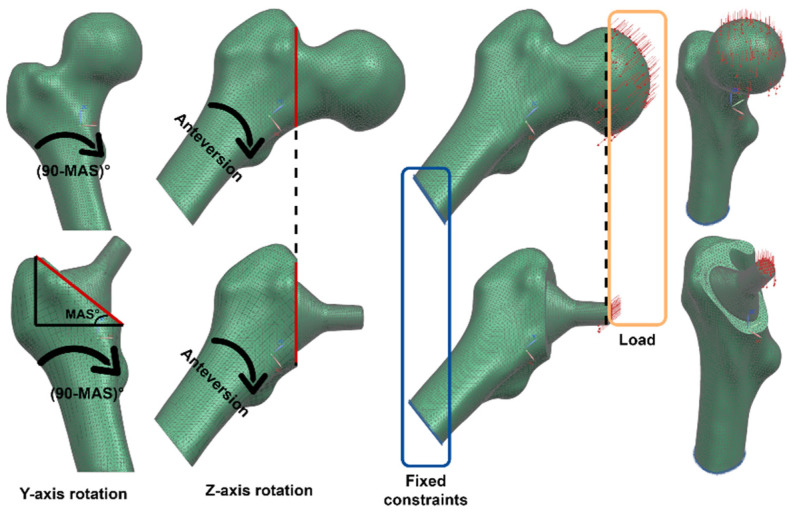
Boundary conditions for the intact and implanted femur.

To apply the load on the intact femur, the body was first placed in a frontal position and rotated (90-MAS)° clockwise with respect to the Y-axis, and then it was rotated at an angle equal to the patient’s anteversion clockwise with respect to the Z-axis, to finally place the load on the cortical femoral nodes that make up the acetabular region—the region located from the beginning to the middle of the femoral head. Since the prostheses were designed using the geometric parameters of the patient, in the implanted femur, the load was applied on the flat part of the receiving taper, which happened to be the same position as in the intact femur—since the cone was designed considering the middle of the femoral head (sphere), the mechanical angle and the anteversion. Therefore, no torque was produced by displacement of the forces, allowing a fair comparison between the intact and implanted femur. In both situations, the movement of the femur was limited through the fixed constraint in the flat part of the cortical and trabecular bone (Figure 10).

Once the meshes were generated, the material and the boundary conditions for each of the bodies were defined, and the simulations were carried out in NX^®^—first for the intact femur of both geometric cases (GC1 and GC2) with the two defined load states (jogging and ISO load), and then for the implanted femur for both geometric cases with the load states and using the two materials (Ti6Al4V ELI and Ti21S) in each of the 3 stems (V1, V2 and V3). Once the results of each simulation were obtained, they were processed to extract the information useful in the evaluation of the SS.

#### 2.5.5. Postprocessing

According to Wolff’s law [76], the adaptation of the bone to the mechanical stimulus causes the remodeling process. However, according to the definition of the mechanostat [77], bone adapts towards a target strain; hence, osteocytes sense this stimulus and send biochemical signals that activate cellular action to remodel bone. In vitro or in vivo studies even use strain gauge rosettes to quantify the strain of the femur and study the relationship between in vivo loading and bone adaptation. Strain data recorded in extensometer studies are usually summarized in terms of principal strains. Therefore, it is necessary to represent the multiaxial strain state as an equivalent metric, i.e., to reduce the complicated and directionally specific strain state to a scalar quantity that is independent of direction. This metric is called “equivalent strain”; it was first introduced by Mikić and Carter [78] with the aim of incorporating strain gauge data in the context of bone adaptation models. Turner et al. [79], through clinical testing of patients with femoral prostheses, evaluated changes in their bone density and found that it adequately modeled bone remodeling. It is easy to interpret, direction-invariant and a positive scalar, because, mathematically, it is the norm of the strain tensor ($\varepsilon_{ij}$):

(22) $$\varepsilon_{ij}=\left[\begin{array}{ccc} \varepsilon_{x} & \varepsilon_{xy} & \varepsilon_{xz}\\ \varepsilon_{xy} & \varepsilon_{y} & \varepsilon_{yz}\\ \varepsilon_{xz} & \varepsilon_{yz} & \varepsilon_{z} \end{array}\right]=\left[\begin{array}{ccc} \varepsilon_{1} & 0 & 0\\ 0 & \varepsilon_{2} & 0\\ 0 & 0 & \varepsilon_{3} \end{array}\right]$$

**Figure 11:**
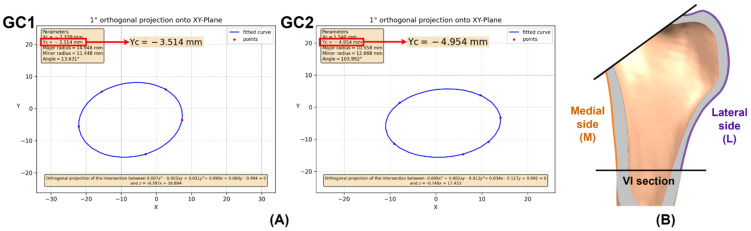
(**A**) Elliptical adjustment of the implantation section. (**B**) Medial and lateral side of the proximal femur.

To perform postprocessing, the proximal femur was cut longitudinally using a plane coincident with the Y-coordinate of the elliptical adjustment performed for the implantation section (I) of each geometric case (Figure 11A). Mimicking the position of the strain gauges and the orthopedist’s analysis of bone density using medical imaging, shielding and bone remodeling over the outer medial (M) and lateral (L) sides of the proximal femur were evaluated using the equivalent strain, in the region bounded by section I and VI (Figure 11B), since these regions undergo more bone resorption in the proximal femur.

## 3. Results and Discussion

### 3.1. Remodeling Curve and Regression Graph

**Figure 12:**
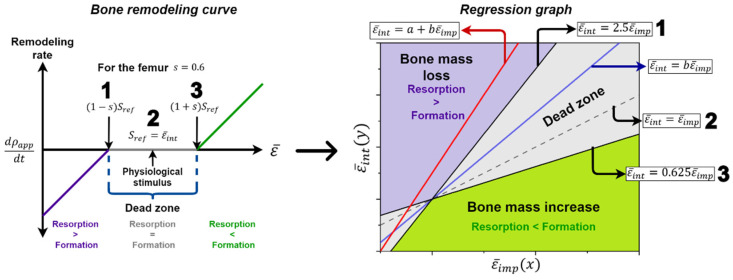
Remodeling curve and regression graph.

The bone adapts towards a target strain, and, if this is greater than desired, the bone mass increases, and if it is less, it decreases. The dead zone is defined as the zone where bone resorption and bone formation are in equilibrium. All these characteristics are summarized graphically in the bone remodeling curve (Figure 12), which relates the variations in apparent density to the mechanical stimulus. Likewise, bone density is directly proportional to stiffness and strength and inversely proportional to its ductility; it is understood that an increase or decrease in density causes undesirable mechanical performance.

Iatrogenic remodeling is related to the bone changes caused by the implant; this type of remodeling should be avoided by the designer and the orthopedist since it contributes to implant loosening and periprosthetic fractures and complicates revision surgeries. Therefore, the ideal stem is one that does not change the femoral biomechanics, does not cause iatrogenic bone remodeling and integrates perfectly through bone ingrowth. However, each stem leads to a specific change in the mechanical response of the femur. Consequently, the designer wants the implant to keep the femur within the dead zone and not cause an excessive increase or decrease in its density. To analyze bone adaptation, the equivalent strain of the mesh element before (${\bar{\varepsilon }}_{int}$) and after (${\bar{\varepsilon }}_{imp}$) stem insertion was obtained; then, the bone remodeling curve was defined, where $S_{ref}$ is ${\bar{\varepsilon }}_{int}$, and in order to establish the dead zone, the “*s*” value was necessary, which, according to the study by Turner et al. [79], is 0.6. Once the parameters were established, the ${\bar{\varepsilon }}_{imp}$ was located on the abscissae to determine whether it was inside or outside of the dead zone.

Despite defining whether or not the femoral region under study is in the dead zone, many designers analyze a region of the femur by averaging the mechanical stimulus of the mesh elements before (${\bar{\varepsilon }}_{int,\;avg}$) and after (${\bar{\varepsilon }}_{imp,\;avg}$) surgery, and calculate the respective strain shielding ($SS_{avg}$). However, the mean of the mechanical stimulus may not represent the loading pattern caused by the stem; consequently, the designer may reach erroneous conclusions using only the average parameters (Table 4). 

Consequently, using concepts related to calculus and statistics, a method was found not only analytically but also graphically to evaluate strain shielding, bone remodeling and femoral biomechanics. This consists of transferring the information from the remodeling curve to a regression graph in an equivalent strain plane of the intact implanted femur, as shown in Figure 12. To obtain the regression graph, an assumption is made: the equivalent strain of the intact femur is dependent on the strain of the implanted femur (${\bar{\varepsilon }}_{int}=f\left({\bar{\varepsilon }}_{imp}\right)$). This may seem contradictory; however, this assumption is very useful because, if a linear regression is performed between the values of the elemental strain before and after the insertion, the result is:

(23) $${\bar{\varepsilon }}_{int}=a+b{\bar{\varepsilon }}_{imp}$$

From this equation, it is possible to obtain the particular designs of femoral stems. For example, the ideal stem, defined as that which fully restores the femoral biomechanics, whose shielding is zero, describes its behavior through Equation (23) when a=0 and b=1. A stem that preserves the femoral biomechanics will be one whose fit results in the Equation (23) with |a|≅0; this means that the strain prior to THR is equal to that after but multiplied by a factor “b”, and the trend in the mechanical response of the femur is maintained in a scaled manner. For this reason, the shielding of this type of implant is:

(24) $$SS=\frac{{\bar{\varepsilon }}_{int}-{\bar{\varepsilon }}_{imp}}{{\bar{\varepsilon }}_{int}}=1-\frac{{\bar{\varepsilon }}_{imp}}{{\bar{\varepsilon }}_{int}}=1-\frac{1}{b}$$

The stem altering biomechanics is defined by Equation (23) for values of “a” and “b”∈ℝ, and as a result, the shielding is:

(25) $$SS=1-\frac{{\bar{\varepsilon }}_{imp}}{{\bar{\varepsilon }}_{int}}=\frac{b-1}{b}+\frac{a}{b{\bar{\varepsilon }}_{int}}$$

High values of ${\bar{\varepsilon }}_{int}$ result in a strain shielding equal to Equation (24). Therefore, this expression was used to approximate the shielding caused by stems that do not restore the femoral biomechanics. In the regression graph (Figure 12), the ideal stem is represented by the dashed black line, the stem that restores biomechanics by the blue line and the stem that modifies the mechanical response by the red line. We defined the types of stems from the assumption ${\bar{\varepsilon }}_{int}=f\left({\bar{\varepsilon }}_{imp}\right)$, and it is necessary to bound the dead zone within the graph:

(26) $${\bar{\varepsilon }}_{imp}=\left(1+s\right){\bar{\varepsilon }}_{int}\Leftrightarrow {\bar{\varepsilon }}_{imp}=1.6{\bar{\varepsilon }}_{int}\Leftrightarrow {\bar{\varepsilon }}_{int}=0.625{\bar{\varepsilon }}_{imp}$$

(27) $${\bar{\varepsilon }}_{imp}=\left(1-s\right){\bar{\varepsilon }}_{int}\Leftrightarrow {\bar{\varepsilon }}_{imp}=0.4{\bar{\varepsilon }}_{int}\Leftrightarrow {\bar{\varepsilon }}_{int}=2.5{\bar{\varepsilon }}_{imp}$$

These lines limit the dead zone, which corresponds to the gray area in Figure 12. From this zone, another two are defined: the purple and green indicate the loss and increase of bone mass, respectively. The adjusted R-Square was used to evaluate how good the linear fit of the equivalent strain of the elements is. The definition of this statistical metric is the proportion of the variance of the dependent variable that can be explained by the independent variable or how well the linear fit is able to model the dependent variable from the independent variable, so it is an indirect measure of how dispersed the points are around the fit line. 

The results obtained from the simulations were used for the analysis, with the equivalent strains of the elements of both regions, lateral and medial. The linear adjustment was performed to obtain the “a” and “b” coefficients, the adjusted R-Square and the SS, and to evaluate the response of each femur to the implantation of the customized stems and the influence of the material from which it is made. Then, using scatter plots by regions, areas of the femoral stem that can be optimized in a following work to mitigate shielding were visualized. Finally, equivalent strain maps extracted from NX^®^ were obtained for the intact and implanted femur with the selected stem and material to verify if the analysis in the lateral and medial zone is representative for both femurs. 

### 3.2. Analysis

**Figure 13:**
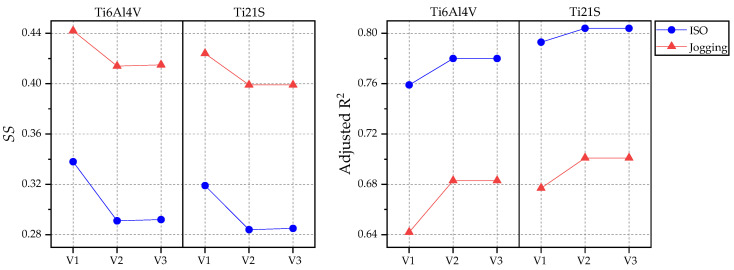
Graphs of the SS and adjusted R^2^ for GC1.

The linear fits between the equivalent strain of the intact and implanted femur with each stem (V1, V2 and V3) were performed for GC1 using both loading states (ISO and jogging) and materials (Ti6V4Al and Ti21S), whose metrics are summarized in Table 5. Figure 13 shows the strain shielding and the adjusted R-Square produced by each stem graphically. In addition, the $SS_{avg}$ was calculated for comparison with the SS obtained from the coefficient “b” of the regression.

The designer is looking for the stem to be as close as possible to the ideal model, with zero shielding, so the implant with the lowest value should be selected. However, as explained in the previous section, the adjusted R^2^ is a statistic that evaluates the goodness of the linear fit, so when it is closer to the unit, it is deduced that the points of the curve present a linear trend and are close to the line, which in turn validates the SS obtained. Therefore, there should be a compromise between the adjusted R^2^ and the shielding. Figure 13 shows that the lowest SS and highest adjusted R^2^ occur when Ti21S is used; regarding implant geometry, the V2 and V3 stems have a very similar mechanical response, with V3 being superior in the SS by thousandths. Then, to select which of the two implants is the indicated one, its volume was evaluated, because the prosthesis with greater volume is heavier, limits the gait and causes patient discomfort. The V2 stem has a volume of 33.25 cm^3^ and V3, 32.368 cm^3^; because V3 is lighter and has metrics similar to those of V2, it is the ideal implant for GC1. 

**Figure 14:**
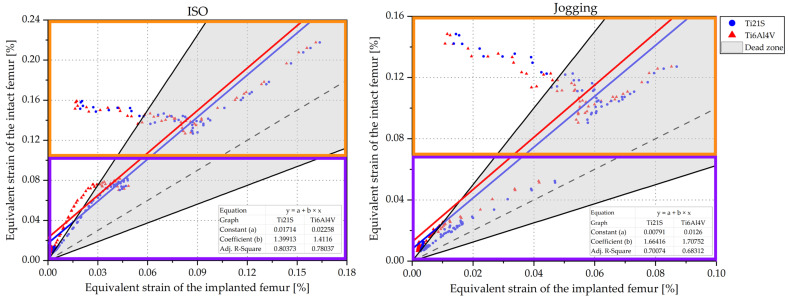
Regression graph of GC1 under ISO and jogging loads.

The influence of the load has not been mentioned, because evaluating either leads to the same conclusion; therefore, to further understand its effect, we plot the response of GC1 to the insertion of the selected stem (V3) when the femur is subjected to the ISO and jogging loads (Figure 14).

Examining the range of the axes, it is perceived that jogging loads the proximal femur less in comparison to the ISO force; this is due to its mechanical nature. The femoral neck fracture is caused by high energy mechanisms such as an axial load on the femur; for this reason, ISO overloads it more. Graphically, the ISO force distributes the load better along the femur; for this reason, the points of its regression graph are more concentrated and follow a linear pattern. On the contrary, the jogging load disperses the points more and causes them not to adapt to the regression; as a result, the adjusted R^2^ is low (Figure 13). Nevertheless, the conclusions obtained by analyzing any of the two load states do not change, i.e., whether examining the femur under ISO or jogging, the same geometry and material is selected. From this perspective, since the use of the ISO force facilitates the testing of prototypes and allows comparison of the experimental results with the finite element analysis, its use is recommended for the evaluation of femoral implants. 

Regression graphs show the influence of the material on the femoral mechanical response. For a closer analysis of its effect, the purple-colored area of the ISO graph in Figure 14 was evaluated.

**Figure 15:**
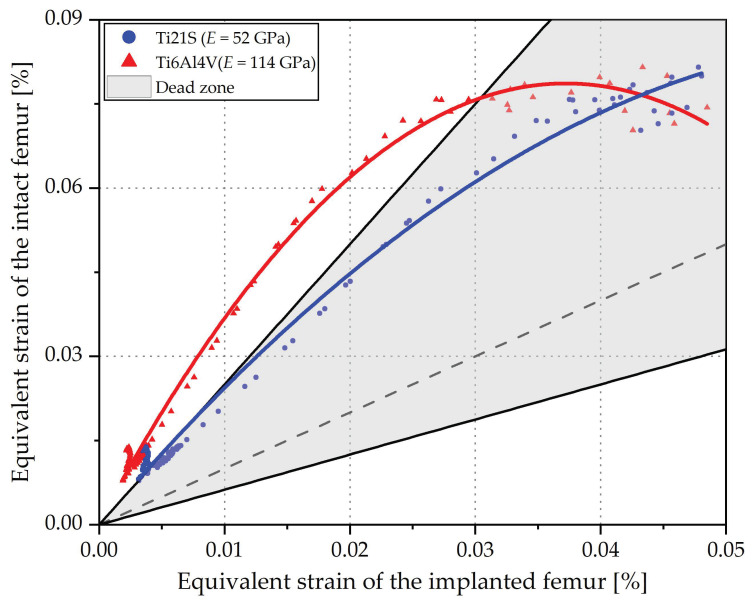
Influence of the modulus of elasticity of the stem material.

Young’s modulus is related to strain shielding. Figure 15 shows that Ti6Al4V, a material with high stiffness compared to the femur, causes greater shielding, and consequently exposes the points to the bone resorption area. 

In addition, due to its quadratic tendency, it alters the femoral biomechanics since it moves away from the linear behavior of the ideal stem and its adjusted R^2^ is lower (Figure 13). In contrast, Ti21S, having lower stiffness, approaches the linear response of the stem that preserves, in a scaled form, the strain of the proximal femur anterior to the THR and maintains the points within the dead zone. In this way, it is verified that, in spite of being the same stem (V3), the selected material originates different mechanical responses; therefore, having a stiffness closer to that of the femur allows the designer to evaluate the behavior originated by the geometry and distinguish it from that caused by the mechanical properties of the material.

**Figure 16:**
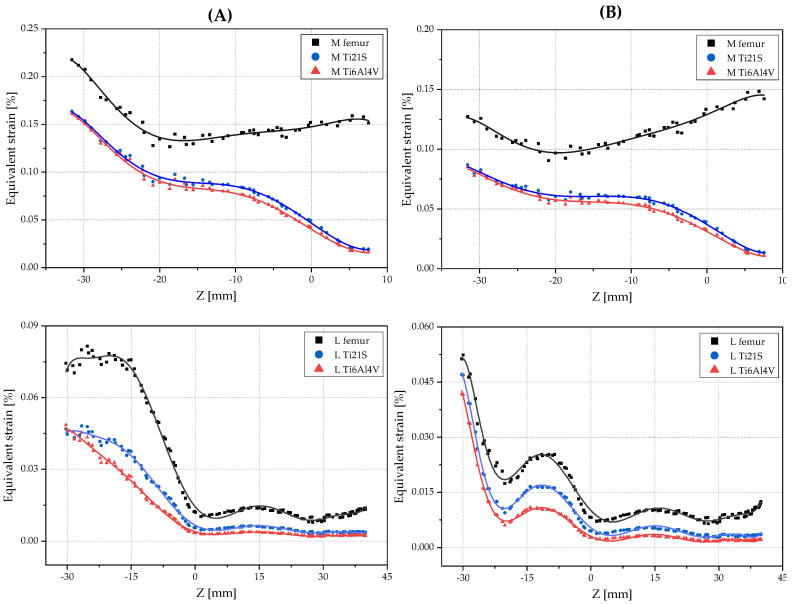
Scatter plots of the equivalent strain of GC1 in the medial and lateral sides under (**A**) ISO and (**B**) jogging loads.

The regression graphs were divided by orange and purple squares enclosing the medial and lateral zones, respectively Figure 14. The medial shows a set of points that follows a negative slope and is outside of the dead zone; the stem is made of either Ti6Al4V or Ti21S. To analyze this behavior in depth, Figure 16 shows the scatter plots of the equivalent strain of the intact and implanted femur subjected to both loading states. 

The plots of the equivalent strain with respect to the Z-coordinate show that the red and blue curves of the implanted femur mimic the black curve that corresponds to the strain of the intact femur, but in the medial part, from Z = −10, the curves of the implanted femur diverge due to the geometry of V3; this section originates the set of points with the negative slope mentioned above.

The scatter plots complement the results obtained from the regression graphs. These plots confirm that the material with the lower modulus of elasticity not only reduces the difference between the strain of the intact and implanted femur, but also preserves the femoral biomechanics. Furthermore, it certifies that any of the loads is useful to select the geometry and material of the stem; further proof of this is that both exhibit the alteration of the medial curve of the implanted femur from Z = −10 onwards. For this reason and due to the above advantages, to study the customized stems of GC2, the femur subjected to the ISO force was evaluated by performing the same analysis of GC1. 

**Figure 17:**
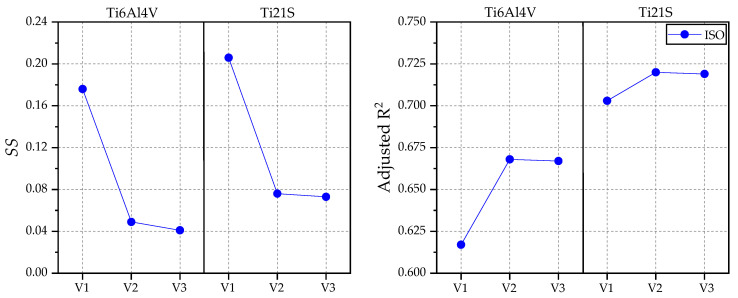
Graphs of the SS and adjusted R^2^ for GC2.

The metrics of the GC2 linear fits are summarized in Table 6, and Figure 17 exposes the SS and adjusted R^2^ produced by each stem graphically.

**Figure 18:**
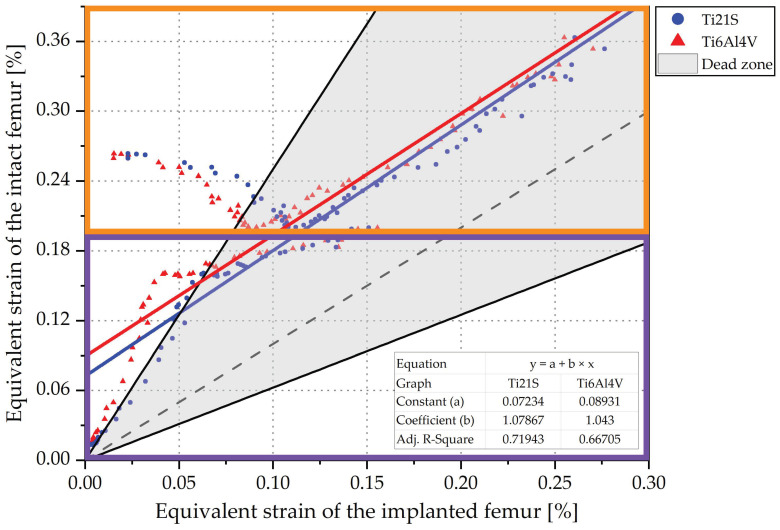
Regression graph of GC2.

Figure 17 shows that the shielding caused by the Ti21S stem is higher compared to those manufactured with Ti6Al4V, which is contradictory to the deductions obtained from the previous analysis. However, the adjusted R^2^ of the Ti21S stem is much higher and, because the shielding is a result of the linear fit, Ti6Al4V cannot be reliably selected as a material in this case. Regarding geometry, again, V2 and V3 have very similar metrics, with the smaller volume being the reason that the V3 stem is preferred. When the metrics do not allow correct selection of the material, the visual method is used. Figure 18 shows the regression graph generated by the chosen geometry, produced with both materials. 

The lateral area of the graph (purple box) shows the influence of material stiffness on the restoration of the femoral biomechanics. It is evident that Ti6Al4V locates a greater number of points outside the dead zone; therefore, its shielding is greater, and the deductions based on the theory and the previous analysis are not contradicted by the information shown in the figure. In short, Ti21S is the ideal material for the fabrication of the customized stem.

The value of the independent term (a) indicates whether the implant deviates from the ideal behavior and alters the load distribution along the femur, which, in this case, is quantified by the equivalent strain; therefore, the farther the regression is from the center of coordinates (|a|>0), the more the implanted femur strain diverges with respect to the intact one, modifying the load received by the bone and increasing the shielding. 

The independent term for GC2 defines that Ti6Al4V more strongly alters the mechanical response of the femur, because the red line is more distant from the center of coordinates. Graphically, the Ti6Al4V regression is above the Ti21S line, cutting the Y-axis at point 0.089. 

**Figure 19:**
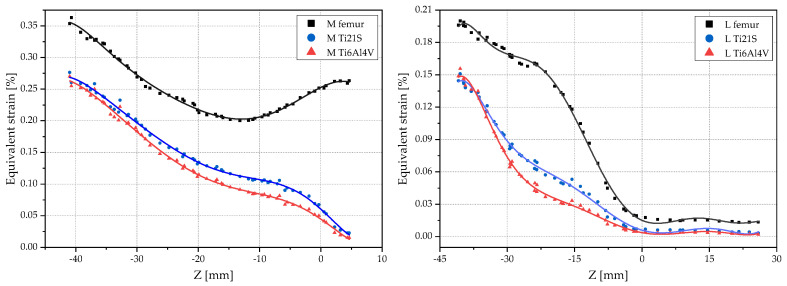
Scatter plots of the equivalent strain of GC2 in the medial and lateral sides.

Figure 18 shows, in the orange box, corresponding to the medial zone, a set of points outside the dead zone and with a negative slope, and, in the purple box, corresponding to the lateral zone, a series of points moving away from the linear trend. The scatter plot of GC2 (Figure 19) supports the choice of material and allows us to identify in which specific regions the geometry of the selected stem should be optimized. In the medial region, it should be improved from Z = −15 onwards, and, in the lateral region, from Z = −30 to Z = −15 because, in these ranges, the strain of the implanted femur, with the stem made of either Ti6Al4V or Ti21S, diverges from the strain of the intact femur, with this effect resulting from the geometry of the V3.

**Figure 20:**
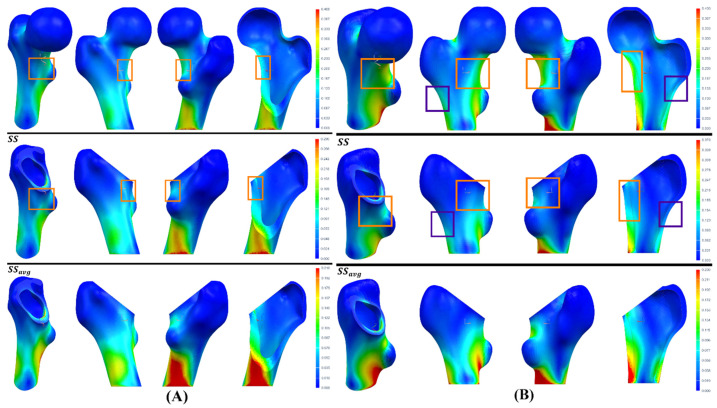
Equivalent strain map of the intact and implanted femur of (**A**) GC1 and (**B**) GC2.

Once the geometry and the material of the customized stem have been selected, it is necessary to verify whether the SS obtained through the proposed method is better than the $SS_{avg}$. For this purpose, we resort to the strain maps, which provide equivalent information to performing photoelastic tests. All the maps of the intact femur of both geometric cases distribute the color scale in the range from 0 to 0.4, while the range goes from 0 to 0.29 and from 0 to 0.21 for the maps of the implanting femur of GC1, and from 0 to 0.37 and from 0 to 0.23 for the maps of GC2 (Figure 20), both calculated from the SS and $SS_{avg}$, respectively, a consequence of the insertion of the V3 stem made of Ti21S.

From the maps, it is verified that the SS adequately quantifies the strain shielding in comparison to the $SS_{avg}$, because of the similarity between the strain maps of the intact and implanted femur when this metric is used. As the femur is mostly within the dead zone, it favors bone ingrowth, which can be supplemented with osteoconductive liners, benefiting the secondary stability, prolonging its lifespan and improving cementless fixation. Likewise, the strain maps certify that the shear planes used for postprocessing (Figure 11), which allow us to study the mechanical behavior and shielding in the lateral and medial part, are a representative sample of the mechanical response of the entire proximal femur. This plane was obtained from the Y-coordinate of the elliptical adjustment of the implantation section, and it reflects another use of the application that aids not only in design, but also in custom stem analysis and selection. The orange and purple boxes show, similar to how the traumatologist evaluates the shielding radiologically, the decrease in the color scale of the medial and lateral region, respectively, which translates into the loss of bone mass of the femur as a natural response to the removal of the neck. This contrasts with the analysis of the scatter plots of each geometric case (Figure 16 and Figure 19).

The study performed by Yan et al. [80], whose boundary conditions are similar to this research, on the shielding caused by two commercial stems—one of a conventional type and the other short calcar-loaded—concludes that the SS in the proximal femur caused by the conventional stem is 0.93 and that by the calcar loading stem is 0.82 approximately. Therefore, both commercial implants place the femur outside the dead zone of the bone remodeling curve, so there will be a bone resorption that, in the long-term, will cause the implant to loosen and a revision surgery will be necessary to replace it. Yamako et al. [81], using strain gauges, quantified, through the equivalent strain, that the shielding in the proximal femur caused by a conventional implant made with Ti6Al4V was 0.61, being positioned at the limit of the dead zone. The shielding resulting from the insertion of the V3 stem made of Ti21S was 0.285 and 0.073 for GC1 and GC2, respectively. Therefore, customization is beneficial in the mechanical response of the proximal femur; this is mainly due to the restoration of the parameters of the patient’s anatomy (neck–shaft angle, anteversion, offset and femoral cavity) and to the selection of a material that has a modulus of elasticity close to the bone. 

However, the precise orientation of the implant is crucial in order not to alter these parameters and consequently its biomechanics; this depends on the surgeon’s expertise, but, to avoid human error in the process, technological assistance is becoming more and more common.

**Figure 21:**
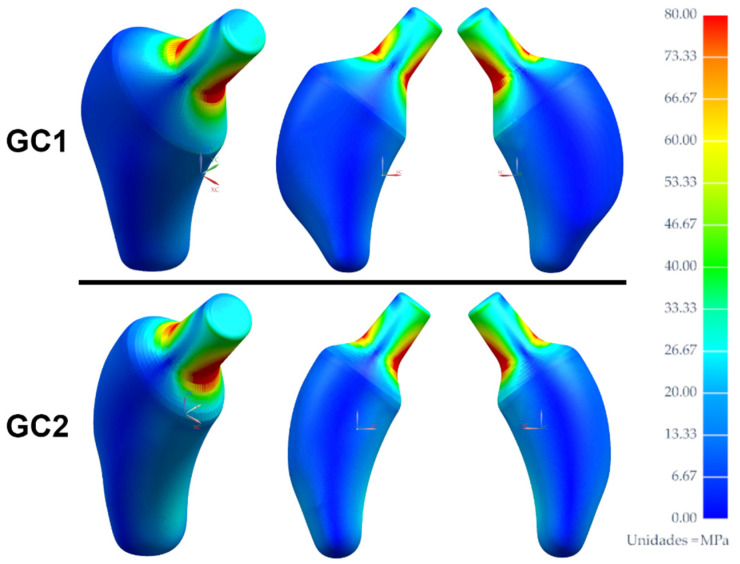
Von Mises stress map of the V3 stem.

Since Ti21S is an isotropic and ductile material, the Von Mises criteria were used. The V3 stem for both geometric cases, subjected to the ISO force, has an average safety factor of 6.055, which guarantees that the implant does not yield to the load. Analyzing the Von Mises stress map of V3 (Figure 21), it is observed that the area with the highest concentration of stresses is the receiving taper; this is beneficial because the stresses generate the compression of the cone walls with the articulating sphere, causing an interference fit and a cold weld between them [82].

**Figure 22:**
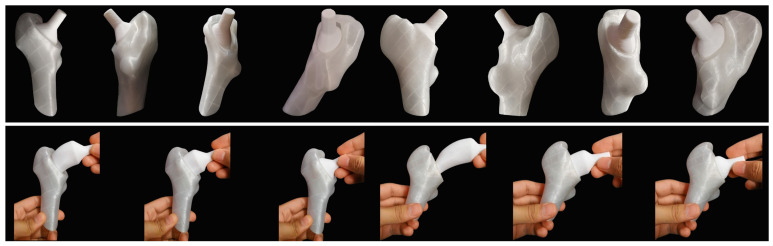
Imitation of the “round the corner” technique for GC1 and GC2.

To verify the implantability of the prosthesis, PLA prototypes of the V3 stem of both geometric cases were made using fused material deposition printing. With the cortical part of the osteotomy already performed, which was used in the *Implantability* (Section 2.4.3), the “round the corner” technique (Figure 6) performed by the traumatologist was imitated when inserting the stem into the canal, verifying that the implant enters normally (Figure 22).

## 4. Limitations and Future Proposals

### 4.1. Limitations of the Study

The authors would like to point out that the study, in its present form, is mainly theoretical and simply aims to present a set of CAD and FEM resources for a more straightforward personalization and in silico evaluation of short stem hip prostheses. Furthermore, the printed prototypes are merely conceptual test probes, in no case intended to be implanted or in vivo tested yet. Several improvements should be performed before considering the proposed designs viable, and the final manufacturing technologies would be completely different to those employed here for a preliminary evaluation of implantability. Probably, for a hypothetical personalized implant with a design such as those presented here, additive manufacturing of metallic alloys (selective laser sintering/melting) would be a good choice, as well as lithography-based ceramic manufacturing using biomedical ceramics, although, in all cases, a final postprocess (sand blasting, PVD-CVD coating) would be beneficial for enhancing osseointegration. The 3D printing of conceptual prototypes helped to initially assess implantability, although design improvements should of course require medical support. In fact, according to the EU Medical Device Regulation 2017/745 (and to most medical regulations worldwide), customized implants cannot reach patients without the prescription and implication in the design procedure of physicians and surgeons. This study provides the basic point of view of biomechanical engineers. 

Collaboration with surgeons would be fundamental for improving the design and for considering challenging issues that can be encountered in real-life surgery, including: (1) the need for rasping and for personalized rasps, which could be designed by downscaling the personalized implants and printing both the customized implants and the supporting tools; (2) the occurrence of unexpected collisions during surgery, which could be alleviated by printing two or three models of the customized design, with slightly different surface finishes or scales, and (3) the potential contraindications when abnormal morphologies are present. In the authors’ opinion, contraindications for the proposed customized designs would be similar to those applicable to short stems in general: presence of hip dysplasia, severe osteoporosis and previous hip osteotomies. 

In any case, before the presented designs can be considered successful solutions, systematic in vitro and in vivo evaluations (with test benches and animal models following the three R principles and applicable regulations) guided by physicians and surgeons are needed. 

### 4.2. Future Research Proposals

Future work, related to the elliptical adjustment application, consists of improving the code and integrating it with a clustering algorithm so that the atypical points of the bone section do not affect the fitting performed by the app. Furthermore, one could consider integrating the program with a computer vision library so that the adjustment is performed only with an image of the cavity (Figure 5B,C), making the extraction of points unnecessary.

For the finite element analysis, one option would be to use the open-source program Bonemat^®^, whose purpose is to define the elastic properties of each element according to the CT information, thus creating a fully anisotropic mesh that will allow a more accurate evaluation of the mechanical response of the femur before and after stem insertion. Using this program, it is no longer necessary to distinguish between cortical and trabecular bone because the mechanical properties are related to the HU information of each CT voxel, thus simplifying the simulation and making it more personalized.

The stem can improve its geometry by manually regulating its oblique sections, the V3 sections being a limiting condition because, if they are exceeded, the new prosthesis will not be implantable. However, it is possible to program and train a machine learning algorithm that, based on an optimization process, determines the best section that preserves the femoral biomechanics and reduces shielding. 

Moreover, topological optimization is an interesting tool that allows a reduction in the weight of the implant and ensures an optimal distribution of the material, as well the optimal load, and it is possible to manufacture it using AM. In fact, the surgeon has the availability of a wide number of prosthesis micro-architectures, thus needing adequate guidelines for the choice of the best one to be implanted in a patient-specific anatomic region [83]. Thus, using strain maps, the designer can improve the stem by mimicking the architecture of the trabecular bone, whose porosity reduces stiffness, decreasing shielding and favoring bone ingrowth, ensuring secondary stability.

A relative micro-displacement analysis should be performed to verify the primary stability, and estimate the secondary stability, of the short prosthesis at the bone–implant interface. In addition, the possibility of designing a short stem that allows the introduction of necessary medications at the postoperative stage should be studied, with the benefits of requiring fewer doses and being applied directly, improving the patient’s recovery and reducing the probability of infection. Likewise, the clinical evaluation of the implanted stems should be extended using surgical assistants such as ROBODOC, because it guarantees the correct cutting and reaming, which allow the precise location of the implant according to the design; in addition, it favors primary stability due to the fact that the tight insertion inside the femoral canal restricts relative displacements, favoring the formation of bone tissue.

## 5. Conclusions

This research work has proposed new tools and concepts that facilitate the custom design of short femoral stems. The application of elliptical adjustment has proven to be very useful for studying femoral morphology, assessing implantability and designing and selecting the customized implant. This instrument can be used in the design of conventional femoral stems or other prostheses in arthroplasty because human error is eliminated when trying to empirically fit an ellipse to the bone cavity.

The implantability has been defined, which is based on the integration of the anatomical parameters with the factors related to surgery, to verify that the stems designed adapt to the femoral cavity and are implantable. Consequently, three geometries for each case study have been designed and evaluated using the finite element method. To analyze the results of the simulations, a methodology based on regression graphs, scatter plots and strain maps that integrate the study of shielding, bone remodeling and femoral biomechanics has been proposed and proven to be more effective and provide more information to the designer compared to the conventional methodology. Based on this, the V3 stem has been selected for having low shielding, keeping the femur within the dead zone and being light in relation to the other geometries, and the Ti21S material, because it restores femoral biomechanics, reduces shielding and does not present the adverse effects of Ti6Al4V related to Alzheimer’s disease and cytotoxicity caused by vanadium.

It has been proven, through analysis, that customized implants restore the patient’s functional mobility, improving their quality of life, because they reproduce the physiological distribution of the femur, in a scaled form, but subsequent optimization is necessary to resemble more closely the mechanical response before surgery. Likewise, it has been shown that the ISO force, being a high energy mechanism, better distributing the load along the femur and facilitating prototype testing and analysis by the finite element method, should be the load used to evaluate the mechanical response of the femur to the insertion of short prostheses. 

## Figures and Tables

**Table 1:** Physical and mechanical properties of the cortical and trabecular bone of each geometric case.

Properties	Cortical Bone		Trabecular Bone	
	GC1	GC2	GC1	GC2
** HU **	1458	1197	779	745
** $\rho_{app}\;\left(g/cm^{3}\right)$ **	1.69	1.41	0.96	0.93
$E_{x}$ (MPa)	9140.76	6534.52	5363.09	4993.1
$E_{y}$ (MPa)	9140.76	6534.52		
$E_{z}$ (MPa)	15,234.61	10,890.87		
$G_{xy}$ (MPa)	3264.56	2333.76	2062.73	1920.42
$G_{yz}$ (MPa)	3808.65	2722.72		
$G_{zx}$ (MPa)	3808.65	2722.72		
** $\upsilon_{xy}$ **	0.4	0.4	0.3	0.3
** $\upsilon_{yz}$ **	0.25	0.25		
** $\upsilon_{zx}$ **	0.25	0.25		

**Table 2:** Mechanical properties of the stem material.

Properties	Ti6Al4V ELI [73]	Ti21S [68]
E (GPa)	114	52
G (GPa)	42.5	19.6
** υ **	0.34	0.33
$\sigma_{y}$ (MPa)	795	709

**Table 3:** Standardized loads for the intact and implanted femur.

	Jogging [74]	ISO [75]
$F_{X}$ (N)	−884.8	**-**
$F_{Y}$ (N)	−15	**-**
$F_{Z}$ (N)	−3222	−2300
$M_{X}$ (Nm)	−0.69	**-**
$M_{Y}$ (Nm)	0.76	**-**
$M_{Z}$ (Nm)	0.09	**-**

**Table 4:** Errors caused by average equivalent strains.

Element	${\bar{\varepsilon }}_{int}\;\left[\%\right]$	${\bar{\varepsilon }}_{imp}\;\left[\%\right]$	SS
1	0.11	0.05	0.545
2	0.075	0.13	−0.733
3	0.5	0.3	0.4
4	0.08	0.1	−0.25
Average	7.875$\;({\bar{\varepsilon }}_{int,\;avg}$)	7.75$\;({\bar{\varepsilon }}_{imp,\;avg}$)	0.016$\;(SS_{avg}$)

**Table 5:** Results for GC1.

		Ti6Al4V			Ti21S		
		V1	V2	V3	V1	V2	V3
**ISO**	**Adjusted R^2^**	0.759	0.78	0.78	0.793	0.804	0.804
	**Constant** (a)	0.024	0.023	0.023	0.017	0.017	0.017
	**Coefficient** (b)	1.511	1.41	1.412	1.468	1.397	1.399
	SS	0.338	0.291	0.292	0.319	0.284	0.285
	$SS_{avg}$	0.574	0.535	0.533	0.496	0.473	0.47
**Jogging**	**Adjusted R** ^2^	0.642	0.683	0.683	0.677	0.701	0.701
	**Constant** (a)	0.013	0.013	0.013	0.008	0.008	0.008
	**Coefficient** (b)	1.792	1.706	1.708	1.735	1.663	1.664
	SS	0.442	0.414	0.415	0.424	0.399	0.399
	$SS_{avg}$	0.605	0.58	0.578	0.525	0.506	0.504

**Table 6:** Results for GC2.

	Ti6Al4V			Ti21S		
	V1	V2	V3	V1	V2	V3
**Adjusted R^2^**	0.617	0.668	0.667	0.703	0.72	0.719
**Constant (a)**	0.097	0.09	0.089	0.073	0.073	0.072
**Coefficient (b)**	1.213	1.052	1.043	1.26	1.082	1.079
** SS **	0.176	0.049	0.041	0.206	0.076	0.073
** $SS_{avg}$ **	0.611	0.512	0.505	0.521	0.443	0.437

## Data Availability

To promote collaborative work and encourage the development and improvement of the proposed design methodology, the STL files of both geometric cases, including their cortical and osteotomized bone, and the short stems are openly available in https://www.thingiverse.com/thing:5187570 (accessed on 3 January 2022).
